# Octahedral Trifluoromagnesate, an Anomalous Metal Fluoride Species, Stabilizes the Transition State in a Biological Motor

**DOI:** 10.1021/acscatal.0c04500

**Published:** 2021-02-17

**Authors:** Mengyu Ge, Robert W. Molt, Huw T. Jenkins, G. Michael Blackburn, Yi Jin, Alfred A. Antson

**Affiliations:** † York Structural Biology Laboratory, Department of Chemistry, University of York, York, YO10 5DD, United Kingdom; ∥ Department of Biochemistry & Molecular Biology, Indiana University School of Medicine, Indianapolis, Indiana 46202, United States; ‡ ENSCO, Inc., 4849 North Wickham Road, Melbourne, Florida 32940, United States; ⊥ Department of Molecular Biology and Biotechnology, University of Sheffield, Sheffield, S10 2TN, United Kingdom; § Cardiff Catalysis Institute, School of Chemistry, Cardiff University, Cardiff, CF10 3AT, United Kingdom

**Keywords:** virus helicase, transition state analogue, ATPase, ^19^F NMR, protein crystallography, general base catalysis, phosphoryl transfer mechanism

## Abstract

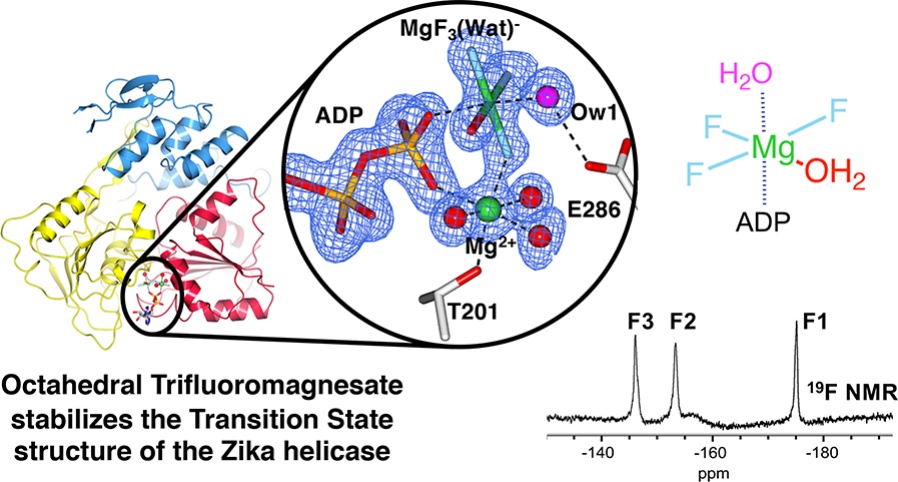

Isoelectronic metal fluoride transition state analogue (TSA) complexes, MgF_3_^–^ and AlF_4_^–^, have proven to be immensely useful in understanding mechanisms of biological motors utilizing phosphoryl transfer. Here we report a previously unobserved octahedral TSA complex, MgF_3_(H_2_O)^−^, in a 1.5 Å resolution Zika virus NS3 helicase crystal structure. ^19^F NMR provided independent validation and also the direct observation of conformational tightening resulting from ssRNA binding in solution. The TSA stabilizes the two conformations of motif V of the helicase that link ATP hydrolysis with mechanical work. DFT analysis further validated the MgF_3_(H_2_O)^−^ species, indicating the significance of this TSA for studies of biological motors.

A central question in discovering the molecular mechanism of a biological machine is understanding how chemical hydrolysis of the nucleotide (e.g., ATP) is coupled with conformational changes that result in mechanical work. This question is usually competently answered by using ATP analogues to stabilize the protein in different conformational states associated with ATP hydrolysis.^1^ Metal fluoride complexes have been immensely useful in such research.^2^ To date, three species of metal fluoride complexes have enabled observation of molecular events that couple the catalytic steps of phosphoryl (PO_3_^–^) transfer to conformational changes by protein crystallography or cryo-electron microscopy (cryo-EM) and by ^19^F solution NMR.^2^ These are tetrahedral BeF_3_^–^ ground state analogues (GSA), octahedral AlF_4_^–^ transition state analogues (TSA) and trigonal bipyramidal (tbp), isosteric MgF_3_^–^ TSA complexes.^2,3^

Here we report a previously unidentified TSA, stabilized by bound magnesium fluoride in an octahedral configuration, containing three fluorines and one water molecule in its equatorial plane. It has been found in a 1.5 Å resolution crystal structure of the Zika virus nonstructural protein 3 helicase (NS3h). The nature of this TSA was verified by ^19^F NMR, which additionally enabled direct observation of its formation and conformational tightening in the presence of ssRNA in solution. The octahedral MgF_3_(Wat)^−^ species was structurally validated by density functional theory (DFT) calculations. Significantly, a catalytically important loop in the protein crystal structure of this novel TSA complex is defined in two alternative conformations associated with coupling ATP hydrolysis to RNA translocation,^4^ demonstrating the advantage of this TSA for studying biological motors which is of wider potential. Furthermore, the novel TSA species identified in this study will inform antiviral drug inhibitor design^5−9^ owing to sequence conservation and indispensability of the helicases.^10^

The fluoromagnesate complex of the Zika NS3h mimicking ATP hydrolysis was prepared by addition of ADP, Mg^2+^ and F^–^. ^19^F NMR spectra showed three well-resolved resonances in 1:1:1 ratio (Figure 1). Solvent induced isotope shift (SIIS) values were also measured (Figure S1, Table 1), as SIIS accurately reflects the number and orientation of H-bond donors around each fluorine.^11^ Replacing ATP by GTP resulted in a closely similar ^19^F spectrum, demonstrating the absence of nucleoside specificity (Figure S2). Since only AlF_4_^–^ TSA structures have been reported hitherto for the NS3 helicases,^12,13^ we titrated 1–5 mM Al^3+^ into a sample of the magnesium fluoride complex containing 10 mM Mg^2+^. This resulted in a progressive 5−50% decrease of the three ^19^F resonances and the growth of an aluminum-associated, rotationally averaged peak at −152.1 ppm for the AlF_4_^–^ TSA (Figure 1a). This partial conversion suggests that for NS3h, the fluoromagnesate TSA is of comparable solution stability to the AlF_4_^–^ TSA.^3,14−19^

**Figure 1:**
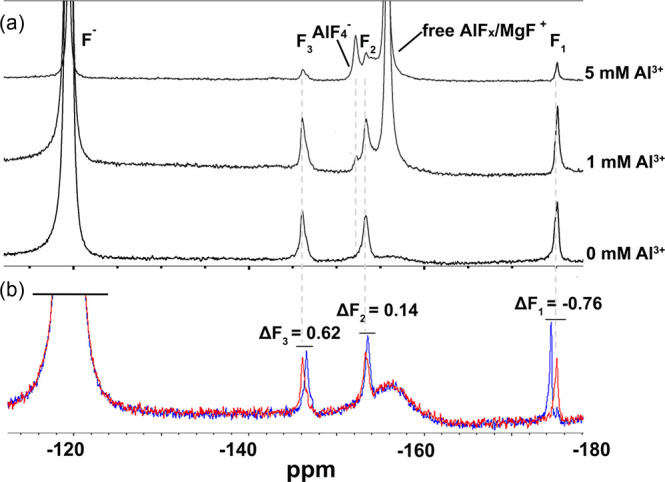
^19^F NMR spectra of (a) ^19^F NMR spectra of the Al^3+^ titration to convert a magnesium trifluoride TSA into an aluminum fluoride complex. (b) ^19^F NMR spectra of ssRNA-free (red) and ssRNA-bound (blue) magnesium trifluoride TSA complexes.

**Table 1:** Chemical and Solvent-Induced Isotope Shifts for ^19^F NMR Signals of RNA-Free and RNA-Bound Zika NS3h MgF_*x*_ Complex

NS3h-MgADP-MgF_3_(Wat)^−^a		F^3^	F^2^	F^1^
RNA-bound	δ^19^F_(90%H_2_O)_	–146.59	–153.58	–174.48
	SIIS	1.40	1.50	0.20
RNA-free	δ^19^F_(90%H_2_O)_	–146.12	–153.36	–175.16
	SIIS	1.38	1.44	0.15

a SIIS = δ ^19^F _(90% H_2_O buffer)_ – δ ^19^F _(100% D_2_O buffer)._

We then investigated conformational changes induced by ssRNA binding^20^ in solution by ^19^F NMR. When ssRNA was added to the magnesium fluoride complex, the three ^19^F resonances changed by only 0.62 ppm (F^3^), 0.14 ppm (F^2^), and −0.76 ppm (F^1^) (Figure 1b). This indicates a relatively small change of the H-bonding network within the NS3h active site and minor conformational changes upon ssRNA binding. Also, the three ^19^F resonances of the complex increase in intensity by ∼20% upon addition of ssRNA, most prominently for F^1^ (Figure 1b), meaning that binding of ssRNA retards exchange between bound and free MgF_*x*_ and results in tighter binding of the TSA complex. In like fashion, a doubling of *K*_D_ for ADP-AlF_4_^–^ in the absence of ssDNA has been observed for Hepatitis C virus (HCV) NS3h by fluorescence polarization.^12^ Binding ssRNA also increases the SIIS values for all three fluorines, reflecting overall H-bond shortening in this TSA complex (Table 1). ^19^F NMR observations thus provide the first direct experimental evidence for structural changes in solution and show holistic, ssRNA-bound, conformational closure of the finely tuned H-bond network around TS phosphate, as also seen for ssRNA-stimulated NTPase activity in HCV NS3h.^20^

The tightening of the active site conformation is also seen in our 1.7 Å resolution crystal structure of the NS3h containing bound MnADP-BeF_3_^–^, which represents a GSA complex (Figure 2a, Table S1). The structure of this complex was obtained by soaking Be^2+^ and F^–^ into NS3h-MnADP crystals (Figures 2a,d). In this structure, the oxygen O^W1^ of the hydrolytic water molecule lies 3.7 Å from Be atom, donating H-bonds to F^1^ (3.1 Å) and to the side-chain C=O of Q455 (2.8 Å) in a prehydrolytic near attack conformation.^16^ These distances are significantly longer than those in an ssDNA-bound NS3h-MnADP-BeF_3_^–^ complex for HCV,^12^ showing that polynucleotide binding for NS3h tightens the pre-TS complex.

**Figure 2:**
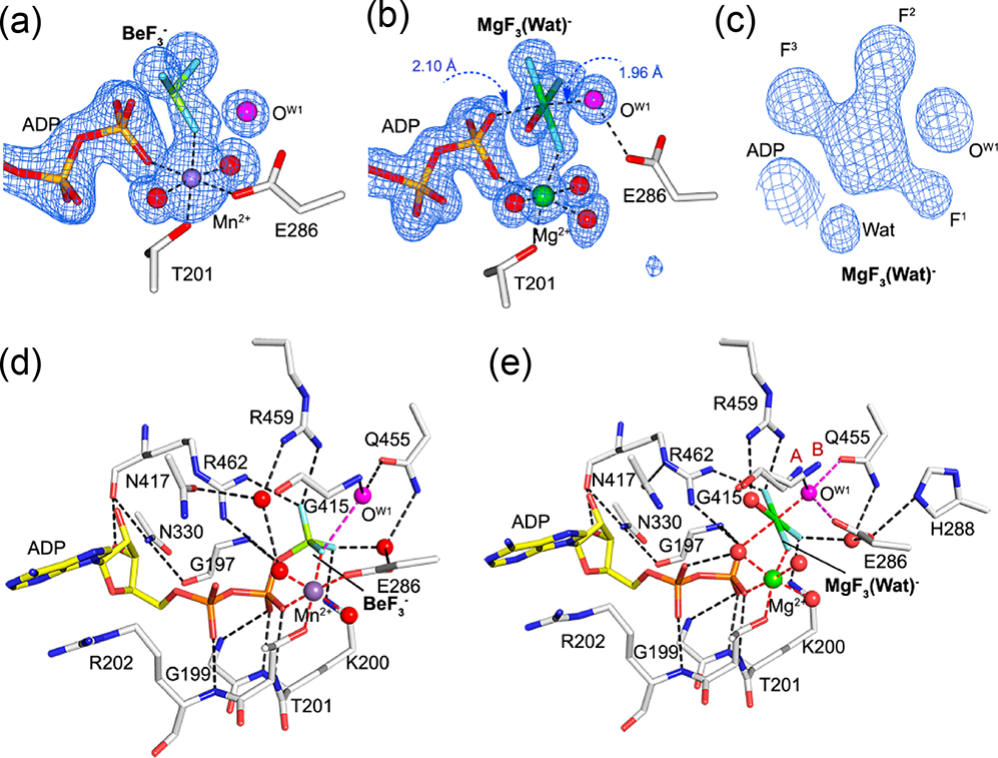
Omit maps (m*F*_o_-D*F*_c_) of (a) NS3h-MnADP-BeF_3_^–^ and (b) NS3h-MgADP-MgF_3_(Wat)^−^ complexes contoured at 4σ and (c) at 8σ. Active site interactions of (d) the NS3h-MnADP-BeF_3_^–^ and (e) the NS3h-MgADP-MgF_3_(Wat)^−^ complexes.

We successfully crystallized the ssRNA-free fluoromagnesate TSA complex of NS3h with bound MgADP (1.5 Å resolution, Table S1). The omit electron density maps clearly defined a square planar species located between the leaving group oxygen O^3B^ of ADP and the hydrolytic O^W1^ (Figure 2b,e). This has not been observed in any of the 24 structures of trifluoromagnesate complexes available in the PDB (Table S2), all of which possess trigonal planar density.^15,21,22^ We repeated the crystallization after adding deferoxamine, a strong aluminum chelator, to exclude potential contamination by aluminum fluoride^14^ and obtained the same crystals. Detailed examination of the omit map of this moiety shows weaker electron density at the site closest to R459 (Figure 2c,e), thereby identifying it as oxygen. In light of the ^19^F NMR analysis, we fitted a water molecule (O^Wat^) into this vertex and fluorines into the other three equatorial vertices to give Mg–F bond lengths refined to 1.88 Å on average and the Mg–O^Wat^ bond length to 2.02 Å, while the axial O^W1^–Mg-O^3B^ angle is 175.6° and ***r***_DA_ is 4.06 Å, characteristic of six-coordinated magnesium^23^ (Figure 2b). This MgF_3_(Wat)^−^ structure explains the chemical shifts and SIISs observed in ^19^F NMR spectra. F^1^ is the most shielded, being coordinated to the catalytic Mg^II^, F^2^ is H-bonded to K200_(NH3+)_ and to a water molecule that is H-bonded to E286 and F^3^ is the most downfield fluorine with two H-bonds from the R459 and R462 guanidinium groups, predicted to neutralize the anionic charge developed on the γ-phosphate during ATP hydrolysis.^24^

The conserved Motif V loop (Figures S3 and S4) in the NS3h-MgADP-MgF_3_(Wat)^−^ complex presents two conformations, A and B (Table S3). Conformation B adopts the “relaxed” position as in the structures of NS3h-MnADP-BeF_3_^–^ (Figure 3a), where the G415 amide is 4.0 Å from water O^W1^ and is H-bonded (3.4 Å) to the backbone carbonyl of E413 (Figure 3b). In conformation A, which shows reorganization of the motif V loop, the G415 amide moves 1.0 Å toward O^W1^, now donating a H-bond (3.0 Å) (Figure 3c). This shows that conformation A participates in TS formation in ATP hydrolysis independently of polynucleotide binding. Motif V is involved in nucleic acid binding;^12,25^ hence, the loop conformation now observed here (Figure 3) shows it can contribute to coupling NTP hydrolysis with RNA translocation. Electron withdrawal from the attacking water O^W1^ by G415 is more than compensated by electron donation from Q445_(C=O)_ and general base E286^26,27^ to complete sp^3^ orbital alignment with the O^3B^-P^G^ antibonding orbital of ATP (Figure S5). Critically, such coordination of O^W1^ orientated by the conformationally flexible loop protects its nucleophilicity from being compromised by adventitious water in a site that is relatively open compared with other NTPases (Figure S6). As we observed in the solution ^19^F NMR, the ssDNA-induced active site tightening is also observed in the transition state (TS) in going from the ssDNA-free Zika NS3h-MgADP-MgF_3_(Wat)^−^ structure to the HCV NS3h-MgADP-AlF_4_^–^ structure (PDB 3KQL)^12^ by 0.1 Å between the oxygen O^W1^ and the side-chain C=O of Q455, and by ∼0.5 Å between the Q455 and E286 side-chains. This tightening seen both by ^19^F solution NMR and by crystallography shows it is independent of crystal packing forces.

**Figure 3:**
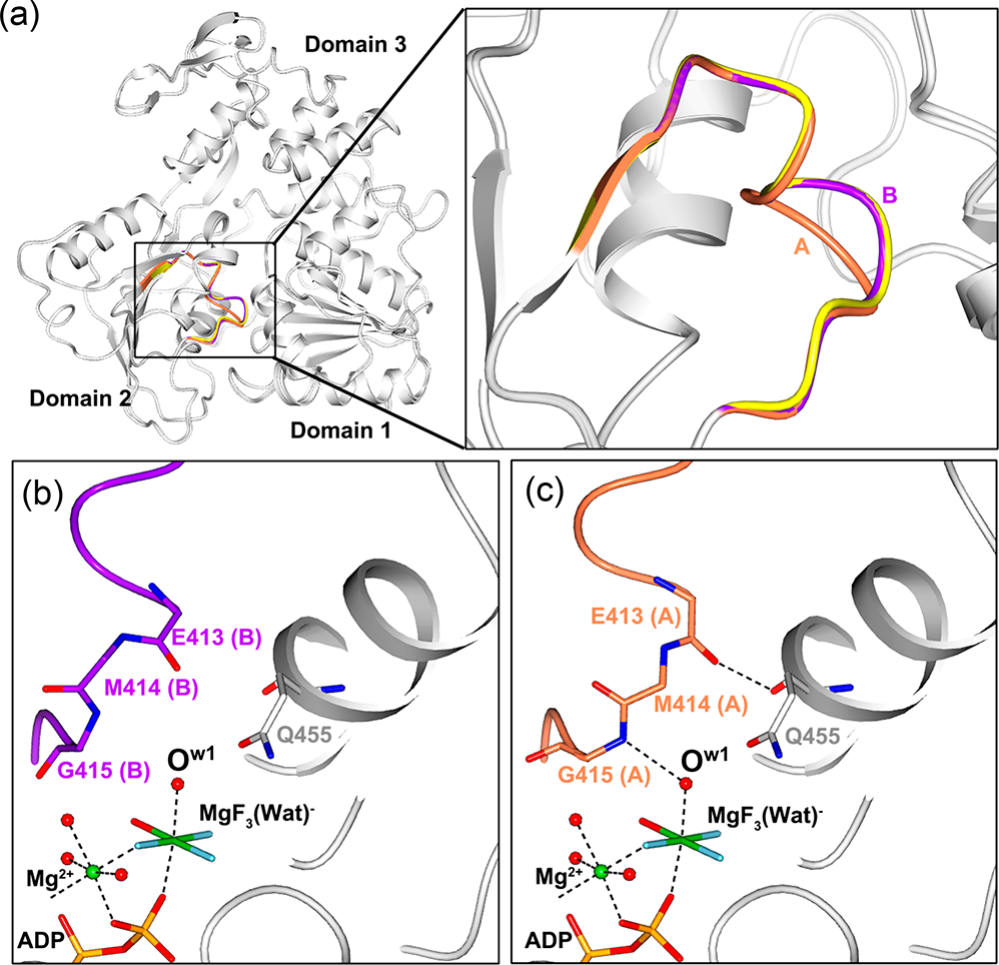
(a) Superposition of the conserved motif V loop conformation A (coral), conformation B (purple) of NS3h-MgADP-MgF_3_(Wat)^−^ structure, and NS3h-MnADP-BeF_3_^–^ (yellow). (b) Loop conformation B (magenta) and (c) loop conformation A (coral) in the NS3h-MgADP-MgF_3_(Wat)^−^ complex structure.

We next analyzed the NS3h-MgADP-MgF_3_(Wat)^−^ TSA complex using DFT by selecting segments from 18 amino acids, representing ADP by MeDP (methyl diphosphate), MgF_3_(Wat)^−^, and nucleophilic H_2_O for the QM zone, a total of 108 heavy atoms (Figure 4, SI).^3,28^ To test the “charge over geometry” hypothesis,^14,17^ both OH^–^ and H_2_O were separately fitted in the position of Wat and established that only H_2_O maintained the octahedral structure seen in the crystal. Similarly, H288 was computed in both its neutral and protonated forms: only neutral H288 delivered the orientation of E286 seen in the crystal structure. The computed NS3h-MeDP-MgF_3_(Wat)^−^ structures for both A and B conformations show excellent agreement with the crystal structure (RMSD 0.30 and 0.40 Å, respectively) (Figure S7a). The network of core H-bonds stabilizing the square planar MgF_3_(Wat)^−^ moiety is well reproduced by six H-bonds from R459, R462, K200, W168, and W331, thus validating the assignment of the electron density to MgF_3_(Wat)^−^ (Figure S7b). Notably, Wat receives a H-bond from R459 guanidinium.^29^

**Figure 4:**
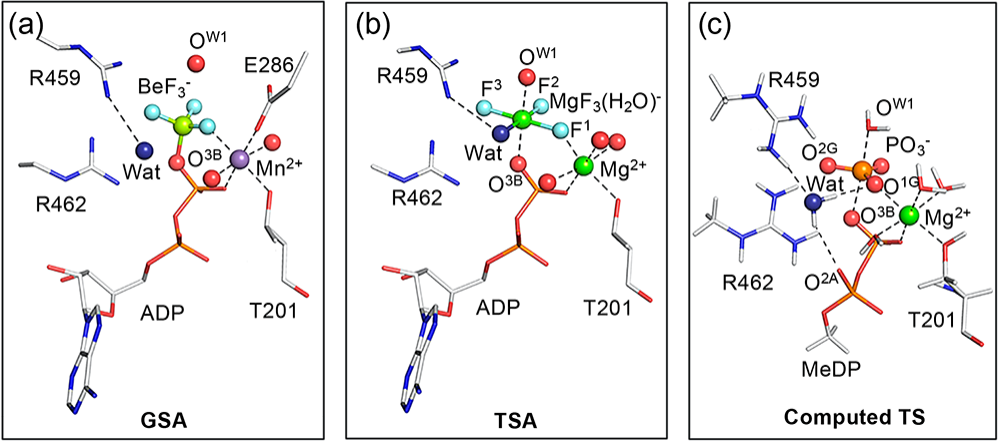
Comparison of water molecule “Wat” in (a) GSA complex for NS3h-MgADP-BeF_3_^–^, (b) TSA complex for NS3h-MgADP-MgF_3_(Wat)^−^ and (c) computed TS for the A conformation in NS3h ATP hydrolysis. The donor O^3B^ (red sphere), Wat oxygen (dark blue sphere), and P^B^/P^G^ (orange) are highlighted.

The QM zone for the TS of ATP hydrolysis by NS3h (Figure 4c) was created by replacing the MgF_3_(Wat)^−^ core by a PO_3_^–^ group and an isolated O^Wat^ (Figure 4b, Table S4). Vibrational frequency analysis showed that a reliable geometry for this computed TS for phosphoryl group transfer was achieved both for conformations A and B (Movies S1, Figure S8). Critical for the reaction mechanism, O^W1^ is coordinated to Q455 and the general base E286, to which it transfers a proton in the TS (SI Movie). Comparing the observed MgF_3_(Wat)^−^ TSA structure with the calculated phosphoryl TS of conformation A, the only significant differences are the following: First, the structure changes from a square planar MgF_3_(Wat)^−^ for the TSA complex to a trigonal planar PO_3_^–^ for the true TS complex. Second, O^Wat^ in the MgF_3_(Wat)^−^ complex in the TS is liberated and moves 1.5 Å away from P^G^ to become triply coordinated to O^2A^, O^1G^, and R459, which fix it 4.3 Å from the nucleophilic water O^w1^ and thus unable to contribute to or impede catalysis of ATP hydrolysis (Figure 4c). This additional water can also be found in the same location in both our NS3h-MnADP-BeF_3_^–^ complex structure (Figure 4a) and in a high-resolution NS3h-ADP structure.^30^ Our computational analysis thus explains how the passive Wat is captured by the trifluoromagnesate as a sixth ligand transforming into a stable octahedral MgF_3_(Wat)^−^ TSA complex (Figure S9). The uniqueness of this octahedral complex clearly signals the absence of an “additional water” in all high-resolution MgF_3_^–^ tbp TSA complexes of ATPases and GTPases structures^2^ yet examined.

In conclusion, the analysis of molecular details of the conformational switch between ssRNA-free and -bound states, central to the function of NS3h during replication, shows a clear distinction between the RNA-free and RNA-bound TSA complexes that results from subtle, significant differences in H-bonding. The characterization of the same changes by ^19^F solution NMR and protein crystallography proves they are not driven by intermolecular interactions in the crystalline state. While motif V is known to be responsible for RNA binding in other NS3h,^12,31^ our results reveal how ATP hydrolysis can be coupled with mechanical translocation of RNA. This analysis of symbiotic spectroscopic, structural, and computational studies on Zika NS3h has delivered an unexpected identification of a previously unknown octahedral MgF_3_(Wat)^−^ TSA. This fourth species of metal fluoride complex may be more widely discoverable for exploration of the mechanism of enzymes involving NTP hydrolysis with active sites equally open to an additional water. A survey of the 142 protein complexes in the PDB with octahedral AlF_4_^–^ (ligand: ALF) strongly suggests that, for some proteins with a relatively open active site and crystallized with aluminum and fluoride present, the octahedral TSA complex observed may have been mis-assigned as AlF_4_^–^ because the concentration of Al^3+^ in the crystallization conditions was inadequate and/or especially ineffective when the solution pH was above 7.5.^14^ The poorly defined TSA electron density in several low-resolution X-ray structures (e.g., 6HEG, 6HPU, 5FHH, and 4ESV) also makes the assignment of their octahedral complex as AlF_4_^–^ perilous. It is clear that only ^19^F NMR is able to resolve whether some of these TSA structures in reality are endowed with an octahedral MgF_3_(Wat)^−^ complex. That, in turn, signals the helicase enzyme has space in its active site to host an adventitious water, and therefore might exemplify the “two-water” mechanism that has been contentiously advocated in catalysis for small G proteins.^32^

## Supporting Information Available

Crystallographic data, structure determination and refinement statistics, raw NMR data, and details of computational analyses (PDF)
Movie S1: vibrational frequency analysis for conformation A (MP4)

## Accession Codes

Structural data for the NS3h-MgADP-MgF_3_(Wat)^−^ TSA and NS3h-MnADP-BeF_3_^–^ GSA complexes have been deposited with the Protein Data Bank under accession codes 6S0J and 6RWZ, respectively.
