# Supplementary material for: Octahedral Trifluoromagnesate, an Anomalous Metal Fluoride Species, Stabilizes the Transition State in a Biological Motor
Source: ACS Catal. 2021 Feb 17;11(5):2769–73. doi: 10.1021/acscatal.0c04500 (PMC7944477; doi:10.1021/acscatal.0c04500)
Supplement: Supplementary file 1 — cs0c04500_si_001.pdf [file cs0c04500_si_001.pdf]

Supporting Information

Octahedral Trifluoromagnesate, an Anomalous Metal Fluoride Species, stabilizes the Transition State in a Biological Motor

Mengyu Ge,† Robert W. Molt Jr.,‖,‡ Huw T. Jenkins,† G. Michael Blackburn,⊥ Yi Jin,¬* and Alfred A. Antson†*

† York Structural Biology Laboratory, Department of Chemistry, University of York, York, YO10 5DD, United Kingdom

‖ Department of Biochemistry & Molecular Biology, Indiana University School of Medicine, Indianapolis, Indiana 46202, United States

‡ ENSCO, Inc., 4849 North Wickham Road, Melbourne, Florida 32940, United States

⊥ Department of Molecular Biology and Biotechnology, University of Sheffield, Sheffield, S10 2TN, United Kingdom

¬ Cardiff Catalysis Institute, School of Chemistry, Cardiff University, Cardiff, CF10 3AT, United Kingdom

*Corresponding authors: JinY6@cardiff.ac.uk and fred.antson@york.ac.uk

Experimental Section

Gene cloning, protein expression and purification

The NS3 helicase protein (residues 183 - 617) corresponding to a Zika virus strain isolated in Brazil (NCBI accession number ANC90426.1)[1] was produced using synthetic DNA (GeneArt, Thermofisher Scientific) cloned into YSBL-LIC(-) vector[2]. N-terminal non-cleavable 6 x His-tagged NS3 helicase was expressed in Rosetta 2 (DE3) cells. Cells from an overnight starter culture were used to inoculated LB medium, incubated with shaking at 37 °C until OD_{600nm} 0.6 - 0.8, when isopropyl β-D-1-thiogalactopyranoside (IPTG) was added to a final concentration of 1 mM. The cells were incubated at 16°C for over 16 h before harvesting by centrifugation. Cells were re-suspended in lysis buffer (20 mM Tris pH 7.5, 500 mM NaCl, 30 mM imidazole, 2 mM DTT) supplemented with 0.1 mg/mL lysozyme and the protease inhibitors (0.5 mg/mL leupeptin, 100 mM AEBSF and 0.7 mg/mL pepstatin A) and disrupted by sonication. The soluble protein was purified by HisTrap HP column (GE Healthcare) equilibrated with binding buffer (20 mM Tris pH 7.5, 500 mM NaCl, 30 mM imidazole, 2 mM DTT) and eluted with an imidazole gradient (30 - 500 mM). 6 x His-tagged NS3 helicase protein was further purified by size-exclusion chromatography using a Superdex 200 HiLoad 16/60 column (GE Healthcare) equilibrated with buffer of 20 mM Tris pH 7.5, 300 mM NaCl, 2 mM DTT. After purification, the yield of NS3h is ~ 110 mg (~55 mg/ml) from 10 L cell culture. However, NS3 helicase protein is not stable and tends to precipitate at room temperature.

NMR spectroscopy

^{19}F NMR spectra were recorded at 298 K on a Bruker Avance 500 MHz spectrometer (operating at 470.38 MHz for fluorine) equipped with a 5 mm QXI probe with z-axis gradients. 500 μM NS3 helicase (NS3h) was used in a buffer containing 20 mM Tris pH 7.5, 300 mM NaCl, 2mM DTT, 10 mM $MgCl_2$, 20 mM NH_4F, and 10 mM ADP/GDP, for recording NS3h-ADP-$MgF_3(Wat)^-$ ^{19}F NMR spectra.

For making NS3h-ADP-$MgF_3(Wat)^-$-ssRNA complex, 0.13 mM ssRNA was added into the sample containing 0.1 mM NS3h, 20 mM Tris pH 7.5, 300 mM NaCl, 1 mM TECP, 10 mM $MgCl_2$, 20 mM NH_4F, and 10 mM ADP. Spectra recorded using 1 mM TECP to replace DTT showed no difference. Solvent-induced isotope shifts (SIIS) on the ^{19}F resonances were measured by comparing spectra for samples separately prepared in buffers containing either 10% D_2O or 100% D_2O. SIIS is defined as the chemical shift difference for [δ ^{19}F (90% H_2O buffer) - δ ^{19}F (100% D_2O buffer)].

The ^{19}F NMR spectra of the Al^{3+} titration experiment to convert NS3h-MgADP-$MgF_3(Wat)^-$ into NS3h-MgADP-AlF_4^- complexes were carried out by addition of 1 mM $AlCl_3$ or 5 mM $AlCl_3$ into a sample of NS3h-MgADP-$MgF_3(Wat)^-$. The 152.10 ppm chemical shift of bound AlF_4^- was observed as a rotationally averaged peak in 90% H_2O buffer. Higher (>5 mM) concentrations of Al^{3+} cannot be achieved, given the severe precipitation of protein in the NMR tube caused by acidification resulting from $AlCl_3$ addition.

Supporting Information

Crystallization

Crystallization was performed by sitting-drop vapor-diffusion using 96-well MRC crystallization plates (Swissci AG). The NS3h-ADP complex crystals (for following soaking and micro-seeding) were grown in 20% w/v glycerol, 10% w/v polyethylene glycol 4000, 0.1 M MES/Imidazole pH 6.5, and 20 mM of each of following salts: sodium formate, ammonium acetate, trisodium citrate, and sodium potassium L-tartrate. The crystal of NS3h-MnADP-BeF_3^- complex was obtained by soaking the NS3h-ADP complex crystals in a solution of 2 mM $MnCl_2$, 2 mM $BeCl_2$, 4 mM NH_4F, 2 mM ADP, 20% w/v glycerol, 10% w/v polyethylene glycol 4000, 0.1 M MES/imidazole pH 6.5, and 20 mM of each of following salts: sodium formate, ammonium acetate, trisodium citrate, and sodium potassium L-tartrate. Crystals were fished out from original drops and soaked in soaking solution for 5 min before being flash-frozen in liquid nitrogen.

The NS3h-MgADP-$MgF_3(Wat)^-$ complex was pre-formed by mixing 5 mg/ml NS3h, 20 mM ADP, 40 mM NH_4F, 0.3 M $MgCl_2$ and 2 mM TECP. Sample mixture was incubated at rt for 30 min before setting-up crystallization trials. Micro-seeding was used to obtain better diffracting crystals, with the seed stock made of the NS3h-ADP complex crystals: 150 nl protein sample mixture was further mixed with 50 nl crystal seed stock and 100 nl reservoir solution, and the crystallization drops were equilibrated against 54 μl of reservoir solution at 20 °C. The complex crystals of NS3h-ADP-$MgF_3(Wat)^-$ were grown in crystallization condition of 15% PEG3350, 0.16 M sodium citrate, and 9% ethylene glycol.

To further prove there was no contamination of Al^{3+} during the crystallization trials, crystals were reproduced from the original crystallization condition with the supplement of 10 mM deferoxamine. X-ray data were collected to compare with that of crystals from the original conditions and showed no geometry or density differences between the original crystals and the new crystals.

Data collection, structure determination and refinement

X-ray data were collected at Diamond Light Source (Didcot). X-ray data of NS3h-MnADP-BeF_3^- complex structure was processed with XDS[3], and X-ray data of NS3h-MgADP-$MgF_3(Wat)^-$ complex structure was processed with DIALS[4-5]. Crystallographic calculations were performed using the CCP4 suite of programs[6]. The NS3h structure was solved by molecular replacement with Phaser[7] against the structure of a DENV NS3h (2JLQ[8],) as a search model followed by automated rebuilding with ARP/wARP[9]. The monomer descriptions for BeF_3^- and $MgF_3(H_2O)^-$ were generated by JLigand[10] with further modification based on previous description[11-14]. The defined ligand dictionary of ADP was created by Grade[15]. The final monomer descriptions of ADP-BeF_3^- and ADP-$MgF_3(H_2O)^-$ were generated by LIBCHECK in CCP4 program Suite[16]. The model was further improved through alternate cycles of manual rebuilding with Coot and refinement with Refmac5[17-18] using isotropic B factors for NS3h-MnADP-BeF_3^- complex structure, and using anisotropic B factors for NS3h-ADP-$MgF_3(Wat)^-$ complex structures in CCP4i2[19]. The $MgF_3(Wat)^-$ ligand is fitted with a defined Mg-F bond length 1.985 Å and Mg-O bond length 2.069Å to give final Mg-F bond lengths of 1.86 Å, 1.86 Å and 1.92 Å and a final Mg-O bond length of 2.02 Å. Figures were generated by the program CCP4MG[20] and PyMOL[21].

Supporting Information

Sequence alignment

Sequence alignment was performed with Clustal Omega[22]. Identities of the aligned sequences were searched by HHPred[23]. Graphic representation was performed with ESPript[24].

Quantum Mechanics (QM) calculation

Our computational model included residues which reproduce the H-bonding network that stabilize the ATP hydrolysis. This also includes all second-shell H-bond recipients/donors to orientate the first-shell H-bond donors. These include L194, H195, P196, G197, A198, G199, K200, T201, R202, R203, D285, E286, H288, M414, G415, Q455, R459, R462, Wat168, Wat169, Wat170, Wat331, Wat320, and ADP.MgF_3Wat^-.

Our electronic Hamiltonian was solved using the KS-DFT[25-26] functional M06-2X[27], known to reproduce molecular geometries very accurately broadly[28] as well as specifically for GTPases[29-30]. All fundamental physical constants are taken from the 2016 CODATA standards[31]. The basis set was chosen per atom, given the need to give adequate electron density flexibility on more important atoms balanced with computational costs. By default, each atom had the basis vectors of cc-pVDZ[32]. Diffuse functions were added (aug-cc-pVDZ[33]) for all atoms which would be formally assigned a negative charge. This includes all of the oxygens on the ATP phosphates and the two carboxylates (D285 and E286). We also placed diffuse functions on all crystallographic water oxygen atoms, as the changes in ligands between the fluoride crystal structure and "real" oxygens are paramount to this paper. For the same reasoning, we also included diffuse functions on the ligand T201 oxygen atom. Due to the high charge density around the carboxylates, we also placed diffuse functions on the carbons joining the two oxygens in D285 and E286. We placed diffuse functions on the nitrogen of H288 which is closest to E286, should it be a recipient of the high charge density of E286 to some extent. Given the consideration of various possible histidine protonation states in this work, we sought to maximize the accuracy of this aspect of the calculation by computing both cationic and neutral imidazole moieties. In the calculations for NS3h-MgADP-$MgF_3(Wat)^-$, all fluorines had diffuse functions. All magnesium and phosphorus atoms were assigned cc-pVTZ for extra polarization potential, given the importance of eclipsed/staggered orientations of ATP.

All reference determinants were considered converged to within 10^{-6} change in density matrix elements. A Lebedev grid consisting of 99 radial and 590 solid angle points was used in all cases, as implemented in Gaussian09[34]. Converged geometries were defined as having a maximum root mean square (RMS) force on any geometric parameter of less than 1.0×10^{-4} Hartree/Bohr and no single geometric parameter could have a force greater than 3.3×10^{-4} Hartree/Bohr. Visual examination of the structures used Gaussview[35] and PyMOL[21]. Our active site model is necessarily truncated compared to the complete protein. At points in the model where one of our atoms would connect to residues not in the model (always a carbon-carbon bond), we simply substitute a hydrogen atom, as these branch points are chemically inert. They are depicted in Figure S8 in green. Such carbons are fixed in position in the geometry optimization (i.e., forces on that atomic center are forced to be zero), since the true protein would not allow that atom to move to avoid steric clash. Transition state optimization used the Schlegel algorithm[36]. The transition state had several minor imaginary

modes as a consequence of the frozen methyl/methylene, all under 50 cm^{-1}, and all liberations. The only imaginary mode not due to frozen methyl/methylene liberations was the bond formation/destruction step of PG. “Imaginary mode” simply refers to the fact that the computed force constant is negative, indicative of a potential energy surface curvature for a transition state[37]. Schematic representations were generated in ChemDraw Prime 17.0.0 (PerkinElmer Informatics, Inc.).

Figures and Tables

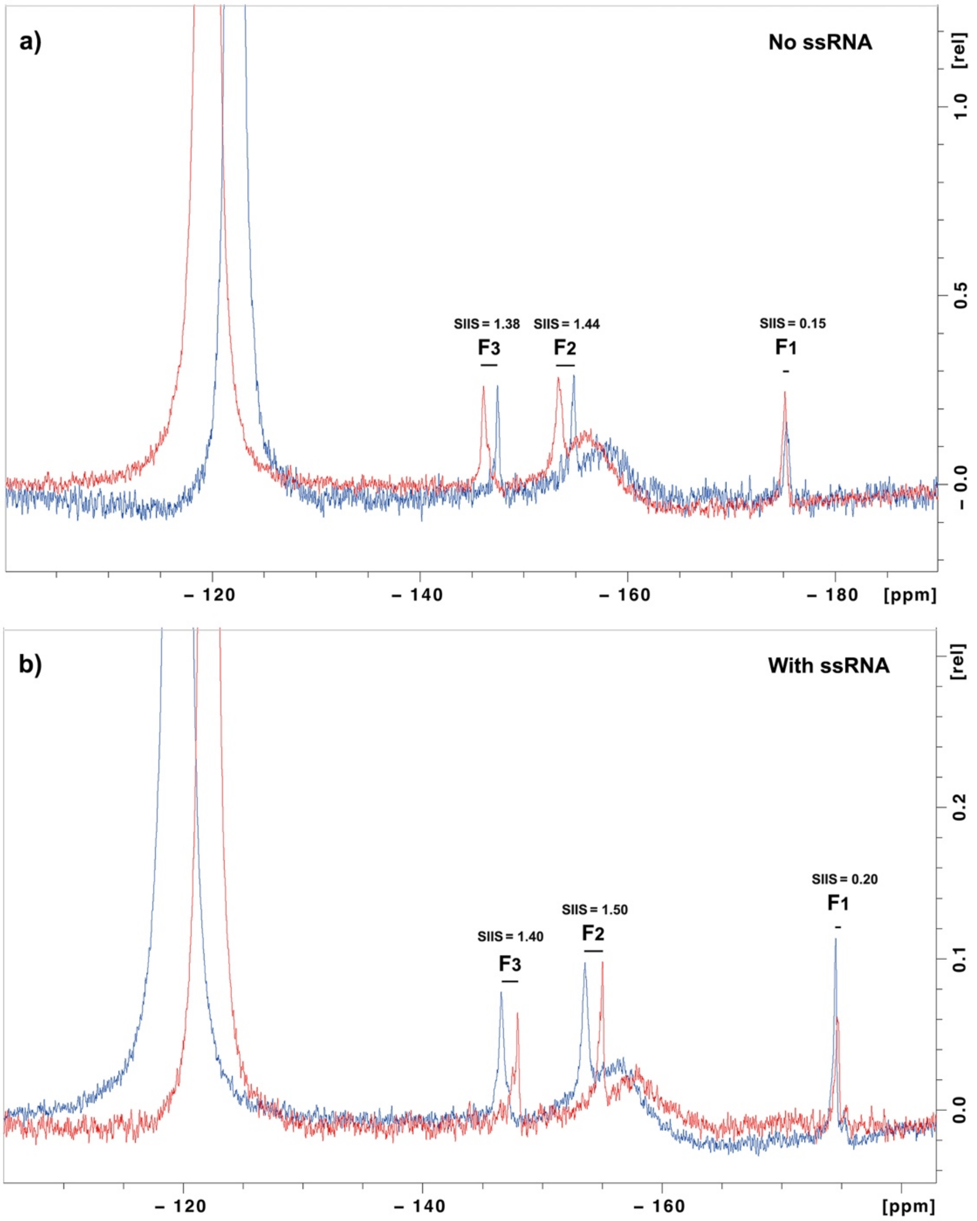

Figure S1. ^{19}F NMR spectra for SIIS measurements on ZIKV NS3h-MgADP-MgF$_3$(Wat)$^-$ TSA complexes in the presence and absence of ssRNA. a) ssRNA-free NS3h-MgADP-MgF$_3$(Wat)$^-$ TSA in 90% H_2O buffer (red; F_3 = -146.0, F_2 = -153.4, F_1 = -175.2 ppm) and in 100% D_2O buffer (blue; F_3 = -147.4, F_2 = -154.8, F_1 = -175.4 ppm). b) ssRNA-bound NS3-MgADP-MgF$_3$(Wat)$^-$ TSA in 90% H_2O buffer (blue; F_3 = -146.6, F_2 = -153.6, F_1 = -175.5 ppm) and in 100% D_2O buffer (red; F_3 = -148.0, F_2 = -155.1, F_1 = -175.7 ppm). Spectra were recorded at 25 °C in buffer containing 20 mM Tris pH 7.5, 0.3 M NaCl, and 2 mM TCEP.

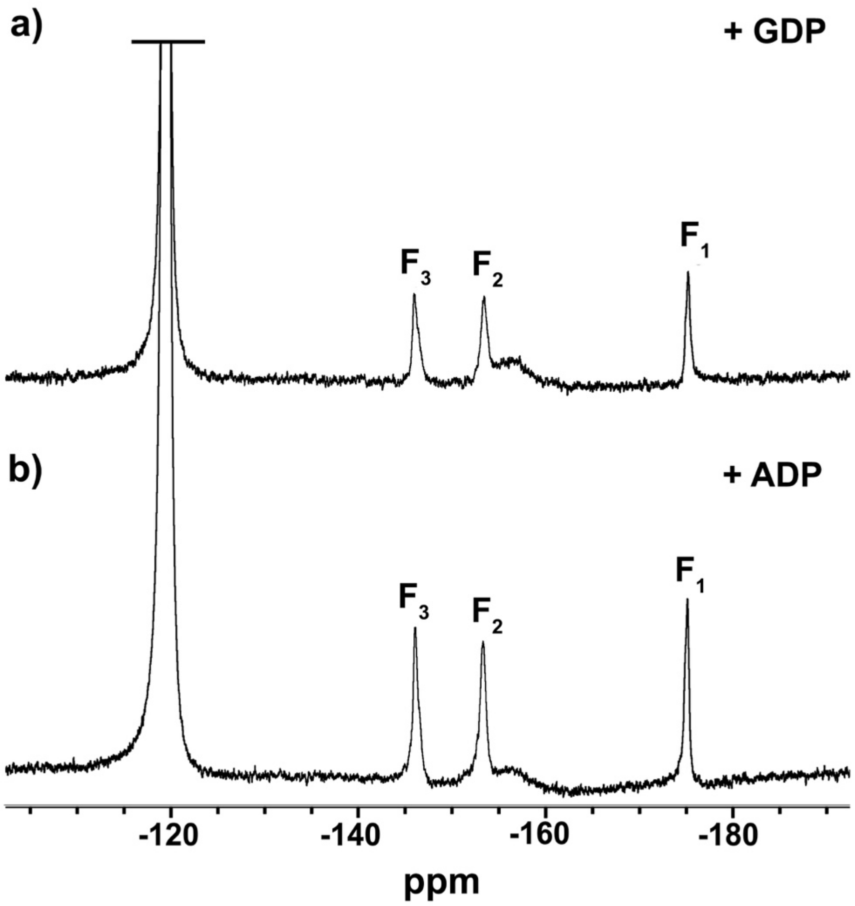

Figure S2. ^{19}F NMR spectra of ZIKV NS3h-MgADP-MgF$_3$(Wat)$^-$ and NS3h-MgGDP-MgF$_3$(Wat)$^-$ TSA complexes. a) NS3h-MgGDP-MgF$_3$(Wat)$^-$ TSA in 90% H_2O buffer (F_3 = -146.0, F_2 = -153.4, F_1 = -175.2 ppm). b) NS3-MgADP-MgF$_3$(Wat)$^-$ TSA in 90% H_2O buffer (F_3 = -146.0, F_2 = -153.4, F_1 = -175.2 ppm). Spectra were recorded at 25 °C in buffer containing 20 mM Tris pH 7.5, 0.3 M NaCl, and 2 mM TCEP.

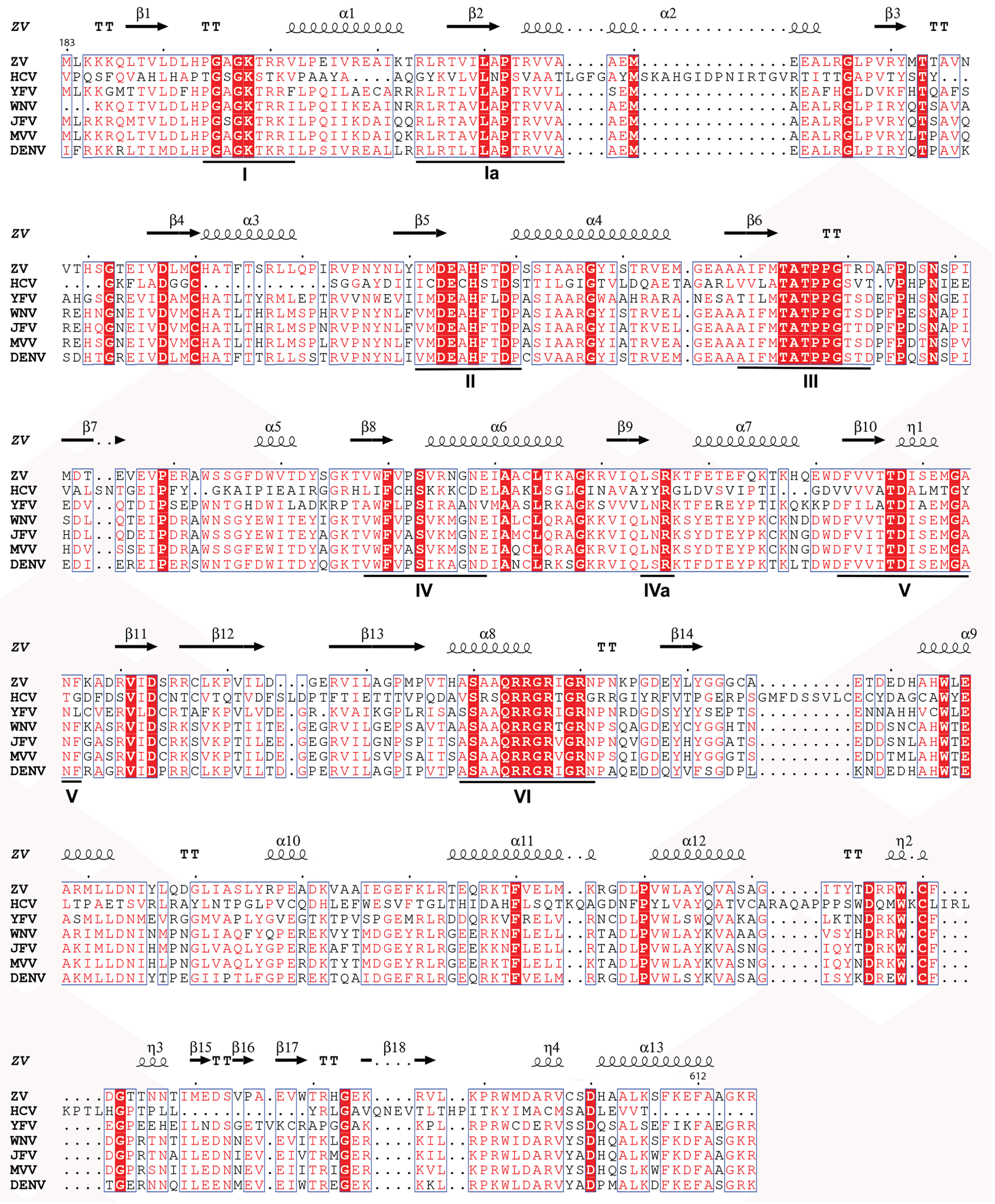

Figure S3. Structure based sequence alignment. Aligned 7 sequences of NS3 helicase from Zika virus (ZIKV, 5mfx), Dengue virus 4 (DENV4, 2JLQ, 71% identity), Yellow Fever virus (YFV, 1YKS, 51% identity), Murray Valley encephalitis virus (MVV, 2WV9, 70% identity), West Nile virus (WNV, 2QEQ, 69% identity), Japanese encephalitis virus (JEV, 2Z83, 66% identity) and Hepatitis C virus (HCV, 3O8B, 26% identity). The conserved motifs I - VI are labelled accordingly.

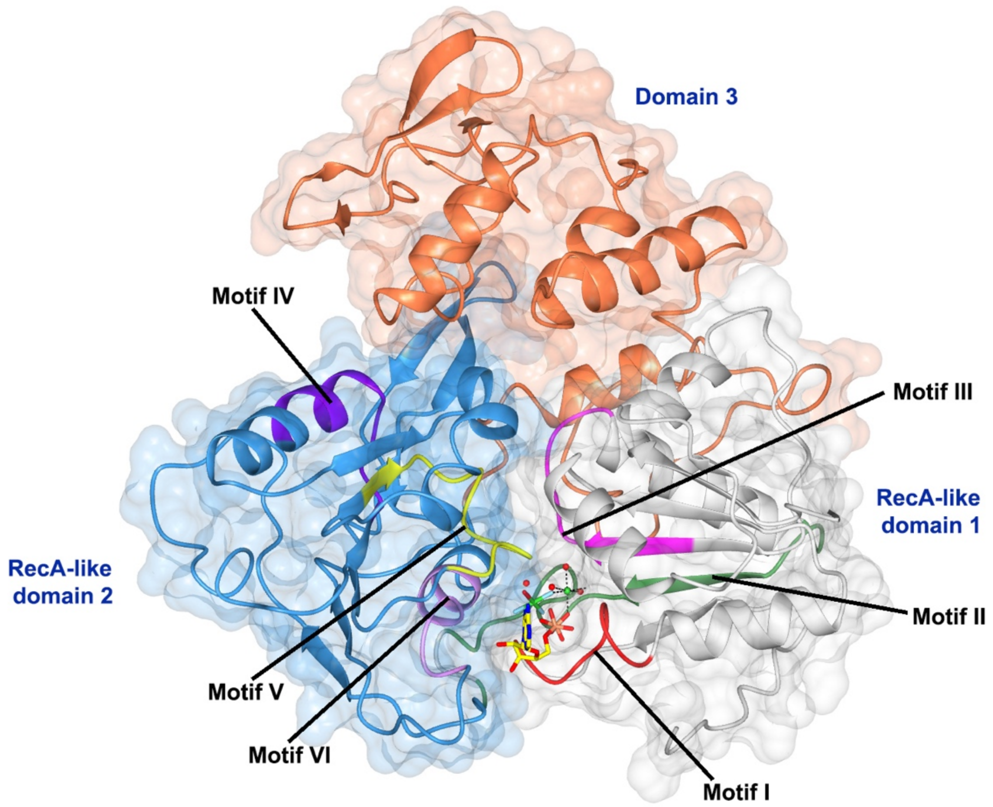

Figure S4. Overall structure of ZIKV NS3h-MgADP-$MgF_3(Wat)^-$ complexes. Domain 1 is in grey, domain 2 is in sea blue, and domain 3 is coral. Conserved motifs have altered colors as indicated. NS3h is shown in ribbon with MgADP-$MgF_3(Wat)^-$ depicted in sticks by CCP4MG[20].

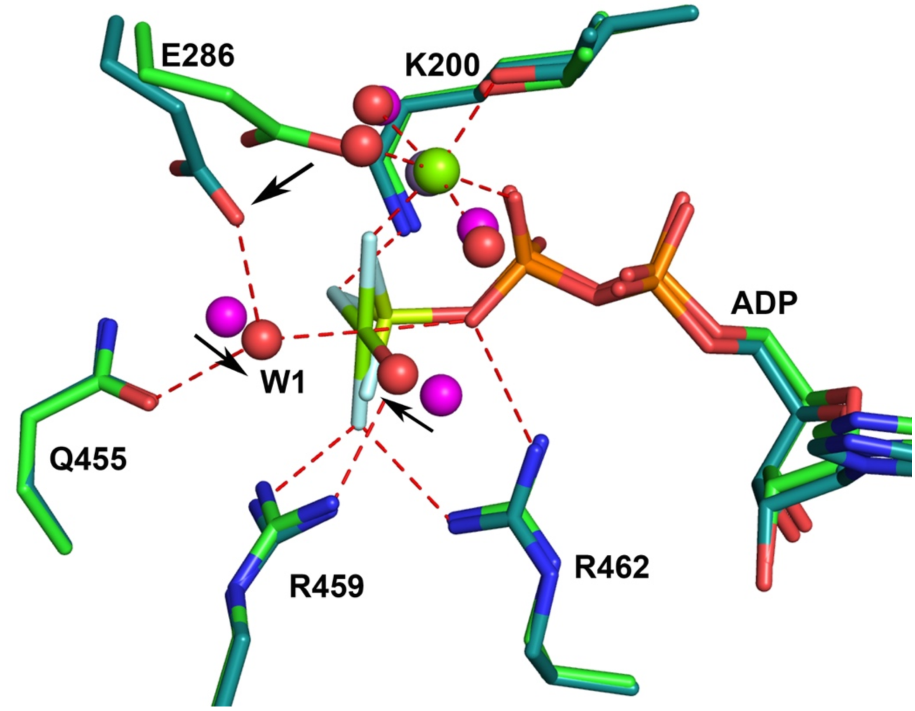

Figure S5. Comparison of ZIKV NS3h-MnADP-BeF_3^- and NS3h-MgADP-$MgF_3(Wat)^-$ complexes to show the coordination change of the general base E286. The NS3h-MnADP-BeF_3^- ground state structure is in light green sticks with waters in magenta. The NS3h-MgADP-$MgF_3(Wat)^-$ TSA structure is in dark green sticks with waters as red spheres, fluorines as light blue sticks.

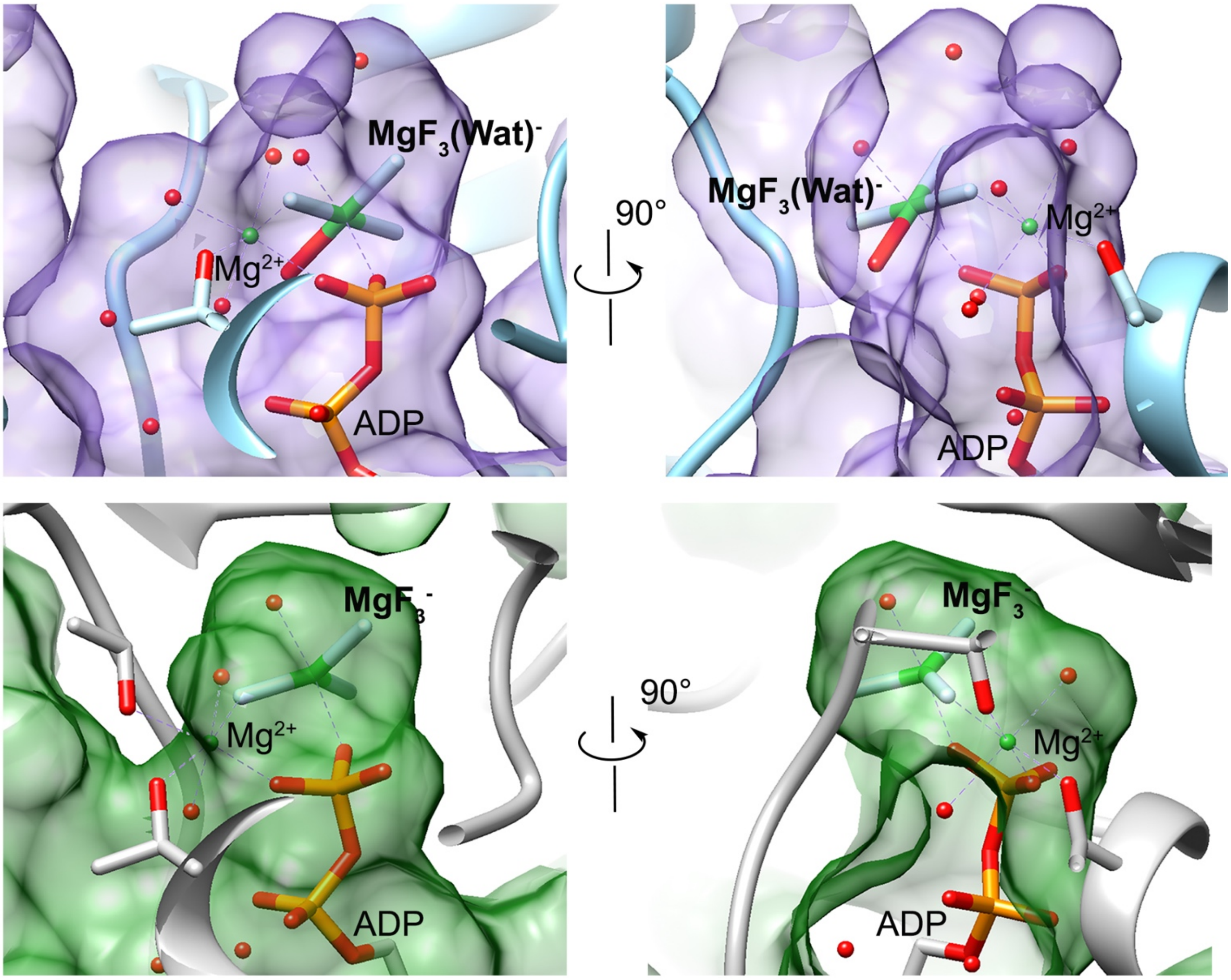

Figure S6. Comparison of the active site pockets for the NS3h-MgADP-$MgF_3(Wat)^-$ TSA complex and RhoA-MgGDP-MgF_3^--RhoGAP TSA complex (pdb 1OW3). Upper panel: The inner surface of the triphosphate binding site highlighted in semi-transparent purple for NS3h-ADP-$MgF_3(Wat)^-$ TSA complex. Protein is highlighted in light blue with $MgF_3(Wat)^-$ shown in sticks. The size of the pocket for binding the TSA and β-phosphate is 256.2 Å^3. **Lower panel:** The inner surface of the triphosphate binding site highlighted in semi-transparent green for RhoA-GDP-MgF_3^--RhoGAP TSA complex. Protein shown highlighted in silver. MgF_3^- moiety is shown as sticks and waters as red spheres. The size of the pocket for binding the TSA and β-phosphate is 69.4 Å^3. The solvent-exclusive surface was calculated with the selected key residues of the ATP binding site in UCSF Chimera[38] (Table S3). The volume of the cavity of ATP binding pocket was measured by UCSF Chimera[39].

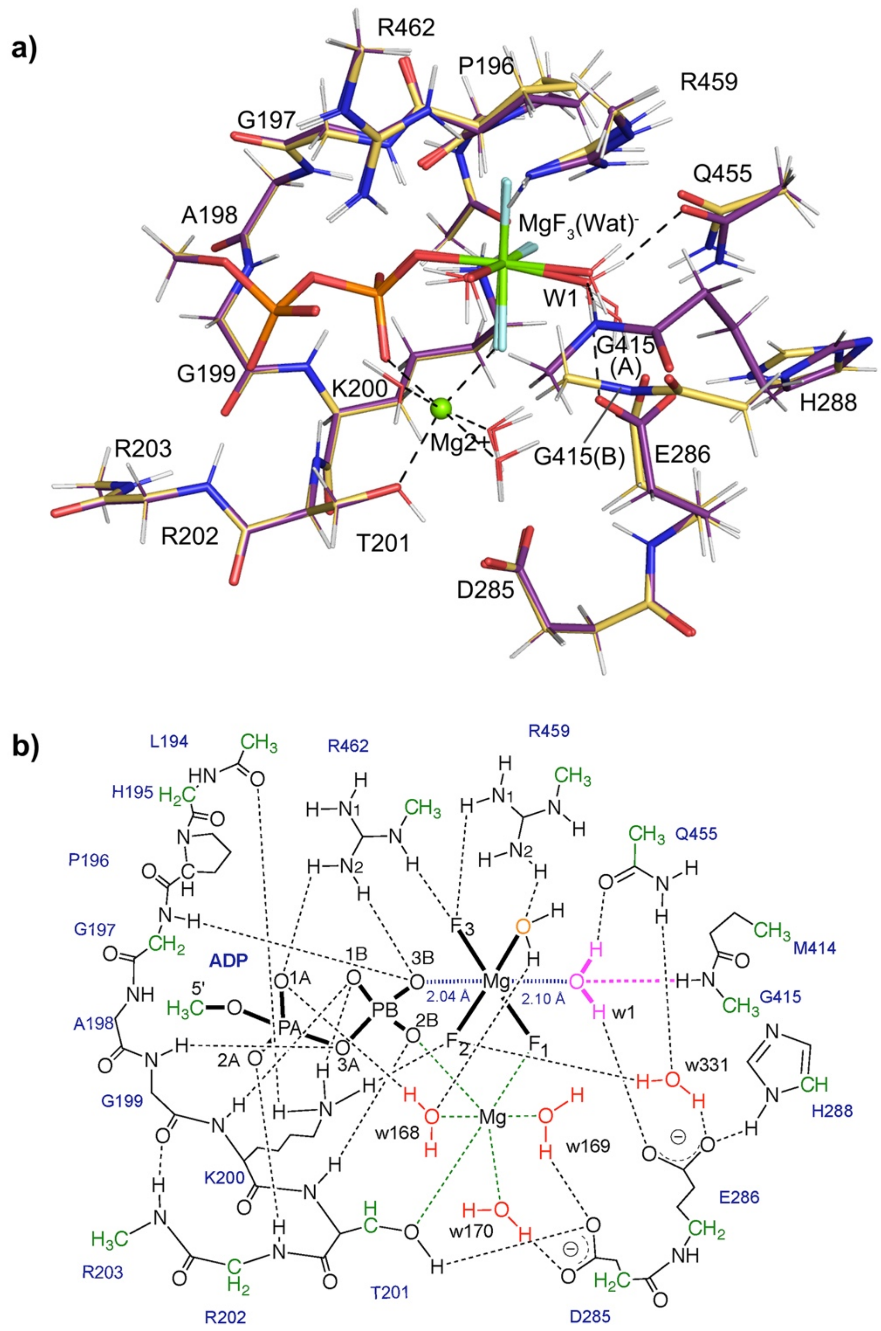

Figure S7. Comparison of the computed NS3h-MgADP-$MgF_3(Wat)^-$ TSA complex for conformers A and B. a) Conformer A is shown in purple, and conformer B is shown in gold. Atom colors: magnesium, green; fluorine, light blue; oxygen, red; phosphorus, coral; hydrogen, gray; nitrogen, blue. Key ionic coordination and H-bonds shown as black dashes. b) Scheme for the QM core used to compute the H-bond network for conformer A of the NS3h-MgADP-$MgF_3(Wat)^-$ complex structure. (H-bonds ≤ 3.20Å, black dashes; nucleophilic water, magenta; structural waters, red; oxygen in the $MgF_3(Wat)^–$, orange).

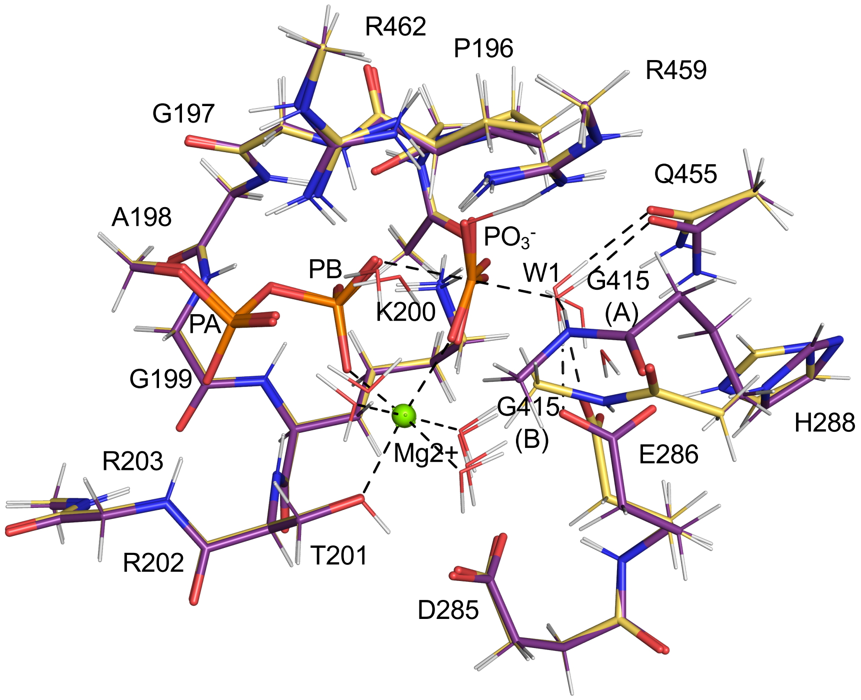

Figure S8. Comparison of the computed NS3h-MgMeDP-PO_3^- TSA complex for conformations A and B. a) Conformation A is shown in purple, and conformation B is shown in gold. Atom colors: magnesium, green; fluorine, light blue; oxygen, red; phosphorus, coral; hydrogen, gray; nitrogen, blue. Key ionic coordination and H-bonds shown as black dashes. The major difference between conformations A and B is, conformation B adopts the "relaxed" position as in the structures of NS3h-MnADP-BeF_3^-, where the G415 amide is 4.0 Å from water O^{W1} and is H-bonded (3.4 Å) to the backbone carbonyl of E413. Whereas in conformation A, which shows reorganization of the motif V loop, the G415 amide moves 1.0 Å towards O^{W1}, now donating a H-bond (3.0 Å)

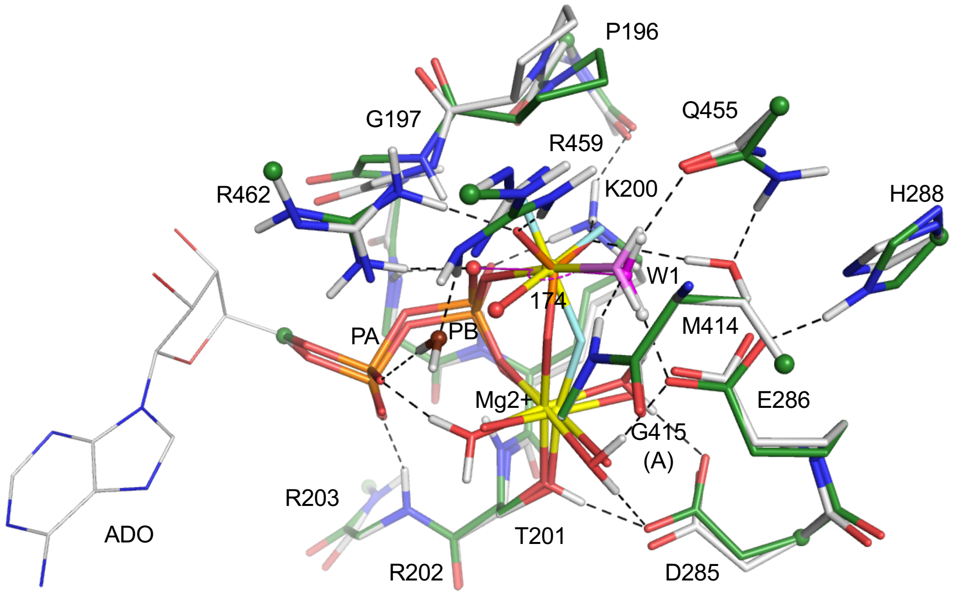

Figure S9. Overlay of the NS3h-MgADP-$MgF_3(Wat)^-$ TSA complex crystal structure with the computed transition state structure for Conformer A. The computed NS3h-MgMeDP-PO_3^- TS is in silver (no hydrogens) and the x-ray structure of NS3h-MgADP-$MgF_3(Wat)^-$ TSA conformer A is in dark green (with polar hydrogens and locked methyls as green spheres). (Atom colors: fluorine, light blue; hydrogen, white; magnesium, lemon; nitrogen, dark blue; oxygen, red; phosphorus, orange; attacking water O4G (spheres), magenta; key H-bonds, black dashes). Comparison of the calculated phosphoryl TS of conformer A with the observed TSA structure shows PG close to the surrogate magnesium (0.13 Å), O1G 0.92 Å from F1, O2G 0.47 Å from F_2, and O3G 0.92 Å from F_3 while the catalytic magnesium has moved 0.34 Å. There is no significant change in the position of any of the amino acid moieties. This computed tbp TS for ATP hydrolysis is somewhat more asymmetric (PG-O4G 1.98 Å and O3B-PG 2.26 Å) than seen for the TSA complex in the crystal structure (PG-O4G 1.96 Å and O3B-PG 2.10 Å), and the O4G-O3B separation is shortened to 4.06 Å. These data establish a concerted TS for ATP hydrolysis with a late TS showing well-advanced P–O bond breaking and bond-making. Most significantly, the nucleophilic water is tetrahedrally organized, having H-bonds with Glu286, Gly415, and $Gln455_{(C=O)}$ and close axial alignment to PG (1.98 Å with in-line angle 174˚). E286 acts a general base catalyst and also orientates the nucleophilic oxygen to direct its sp^3 lone pair at PG, which is in contrast to the anti-catalytic H-bond seen in the NAC structure for the BeF_3^- complex (PDBID: 6RWZ). The proximity of an isolated water in the computed TS (chocolate sphere) having H-bonds with O2A and R459 with the water for $MgF_3(Wat)^–$ in the octahedral crystal complex (red sphere) shows a separation of their oxygens of only 1.5 Å. This suggests that assembly of this octahedral complex may start with a tbp pentacoordinate trifluoromagnesate which, on finding a proximate water only 3 Å from its magnesium captures it (gold arrow), thus generating the octahedral six-coordinate complex which we have found to be very stable. The failure of magnesium in other tbp trifluoromagnesate complexes to capture a water in this fashion is entirely consistent with the regular exclusion of any isolated water from their catalytic cores for phosphoryl transfer.

Table S1. Data collection and refinement statistics.

	NS3h-ADP-BeF_3^-	NS3h-ADP-$MgF_3(Wat)^-$
**Data collection**		
Space group	P 1 2_1 1	P 1 2_1 1
Cell dimensions		
a, *b*, *c* (Å)	52.92, 71.36, 57.35	52.94, 69.60, 57.75
α, β, γ (°)	90, 93.73, 90	90.00, 94.40, 90.00
Resolution (Å)	57.23 - 1.70 (1.74 - 1.70)	69.58 - 1.50 (1.53 - 1.50)
R_{meas} [a]	0.065 (1.508)	0.055 (1.149)
$I/\sigma(I)$ [b]	13.1 (1.0)	10.1 (1.2)
CC(1/2)	0.999 (0.444)	0.998 (0.419)
Completeness (%) [c]	99.4 (94.3)	99.7 (99.0)
Multiplicity	4.1 (4.0)	2.9 (2.9)
**Refinement**		
PDB ID	6RWZ	6S0J
Resolution (Å)	57.23 - 1.70	57.65 - 1.50
No. reflections	44518	63340
R_{work} / R_{free}	0.167/ 0.206	0.145 / 0.189
No. atoms		
Protein	3478	3439
Ligand/ion	32	33
Water	353	463
B factors ($Å^2$) [d]		
Protein	33.6	28.3
Ligand/ion	34.6	31.4
Water	41.7	43.6
R.M.S. deviations		
Bond lengths (Å)	0.013	0.010
Bond angles (°)	1.540	1.586

[a.] $R_{meas} = \Sigma h(n/n\text{-}1)^{1/2}\ \Sigma_i\ |I_i(h) - \langle I(h)\rangle| / \Sigma h \Sigma i I_i(h)$, where $I_i(h)$ and $\langle I(h)\rangle$ mean measurement of the intensity of reflection *h*; [b.] I/σI: Signal to noise ratio for merged intensities; [c.] Completeness for unique reflections, highest resolution bins in brackets; [d.] B factor (temperature factors) averaged for atoms of protein, ligand/ion and water, respectively. *Values of highest resolution shell in brackets.

Table S2. List of MgF_3^- complex structures (PDB ligand entry: MGF).

PDB ID	space group	Protein name	Resolution	Reference
4C4R	$P2_12_12_1$	β-Phosphoglucomutase (βPGM)	1.10 Å	[40]
2WF5	$P2_12_12_1$	β-Phosphoglucomutase (βPGM)	1.30 Å	[41]
5OLX	$P2_12_12_1$	β-Phosphoglucomutase (βPGM)	1.38 Å	[42]
2WZB	$P2_12_12_1$	Human phosphoglycerate kinase	1.47 Å	[43]
4C4S	$P2_12_12_1$	β-Phosphoglucomutase (βPGM)	1.50 Å	[40]
5JAJ	$P2_12_12_1$	LGP2 (RNA binding protein)	1.50 Å	[44]
3MSX	C2 2 2_1	RhoA GTPase	1.65 Å	Unpublished
4NV0	P 1 2_1 1	cytosolic 5'-nucleotidase IIIB (cN-IIIB)	1.65 Å	[45]
5IRC	$P2_12_12_1$	RhoA GTPase	1.72 Å	[46]
4DLC	P 4_3 2_1 2	Trypanosoma brucei dUTPase	1.76 Å	[47]
1OW3	$P2_12_12_1$	RhoA GTPase	1.80 Å	[48]
3T9E	$P2_12_12_1$	Human diphosphoinositol pentakisphosphate kinase 2 (PPIP5K2)	1.90 Å	[49]
3QHW	P 1 2_1 1	CDK2 Kinase	1.91 Å	[50]
3ZOZ	$P2_12_12_1$	Human Phosphoglycerate Kinase	1.95 Å	Unpublished
5OLY	P 1 2_1 1	β-Phosphoglucomutase (βPGM)	2.00 Å	[42]
3QHR	P 1 2_1 1	CDK2 Kinase	2.17 Å	[50]
2IS6	P 1 2_1 1	UvrD helicase	2.20 Å	[51]
5HPY	$P2_12_12_1$	RhoA GTPase	2.40 Å	[52]
5M6X	P 1 2_1 1	RhoA GTPase	2.40 Å	[30]
4BEW	$P2_12_12_1$	Sarcoplasmic/endoplasmic reticulum calcium ATPase 1	2.50 Å	unpublished
5MPM	P 1 2_1 1	Sarcoplasmic reticulum Ca2+-ATPase	3.30 Å	unpublished
4JSV	P 2 2_1 2_1	mTOR kinase	3.50 Å	[53]
4BEV	$P2_12_12_1$	Copper efflux ATPase	3.58 Å	unpublished
3JBZ	-	ATM kinase	28.0 Å	[54]

All 24 entries for the MGF available in the PDB database to May 2019 are listed in the table, 19 entries (resolution better than 2.50 Å).

Table S3. X-ray structures of *Flaviviridae* family NS3 helicases in complex with TSA.

PDB ID	Protein	Resolution (Å)	Space Group	TSA	With RNA/DNA bound	Motif V loop conformation
6S0J (this work)	ZIKV NS3h	1.5	$P2_1$	$MgF_3(Wat)^-$	no	A&B
2JLY	DENV NS3h	2.4	C2	$ADP\text{-}PO_4^-$	yes	B
2JLX	DENV NS3h	2.2	C2	$ADP\text{-}VO_4^-$	yes	A
5E4F	HCV NS3h	2.1	$P2_1$	AlF_4^-	no	B
3KQL	HCV NS3h	2.5	P2	AlF_4^-	yes	A

Comparison of the loop (motif V) conformations of all *Flaviviridae* family viruses NS3h-TSA complexes structures which are available in the PDB by July 2018.

Table S4. Residues selected for the calculation of ATP/GTP β- and γ-phosphate binding pockets.

NS3h-ADP-$MgF_3(Wat)^-$ TSA complex	RhoA-GDP-MgF_3^--RhoGAP TSA complex
**PDB: 6S0J**	**PDB: 1OW3**
Pro196.a	Gly12.b
Gly197.a	Asp13.b
Ala198.a	Gly14.b
Gly199.a	Ala15.b
Lys200.a	Cys16.b
Thr201.a	Gly17.b
Arg202.a	Lys18.b
Val228.a	Thr19.b
Glu231.a	Tyr34.b
Asp285.a	Val35.b
Glu286.a	Pro36.b
Thr316.a	Thr37.b
Ala317.a	Asp59.b
Met414.a	Thr60.b
Gly415.a	Ala61.b
Ala416.a	Gly62.b
Asn417.a	Gln63.b
Gln455.a	Arg85.a
Arg459.a	
Arg462.a	

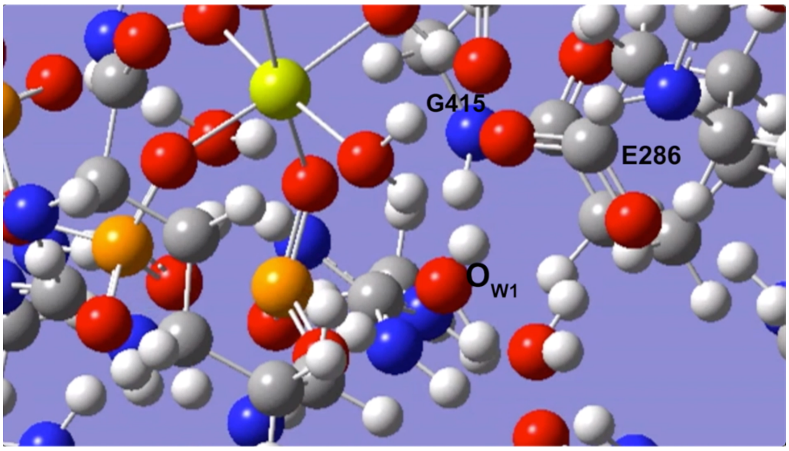

Movie S1. Visualization of the characteristic imaginary vibrational mode of the TS model with motif V loop of conformer A in which the W1 water nucleophile attacks the PG of ATP supported by H-bonding from Gly415. The TS vibrational mode for the complete QM cluster embraces concerted proton transfer from this water to E286 and thus visualizes classical general base catalysis.

References

1. Donald, C. L.; Brennan, B.; Cumberworth, S. L.; Rezelj, V. V.; Clark, J. J; Cordeiro, M. T.; Freitas de Oliveira França, R.; Pena, G. S. Wilkie, L. J.; Da Silva Filipe, A.; Davis C.; Hughes J.; Varjak M.; Selinger M.; Zuvanov L.; Owsianka A. M.; Patel A. H.; McLauchlan J.; Lindenbach B. D.; Fall G.; Sall A. A.; Biek R.; Rehwinkel J.; Schnettler E.; Kohl A. Full Genome Sequence and sfRNA Interferon Antagonist Activity of Zika Virus from Recife, Brazil. *PLoS Negl. Trop. Dis.* **2016**, *10*, 1–20.
2. Fogg, M. J.; Wilkinson, A. J. Higher-throughput approaches to crystallization and crystal structure determination. *Biochem. Soc. Trans.* **2008**, *36*, 771–775.
3. Kabsch, W. XDS. *Acta Crystallogr. Sect. D Biol. Crystallogr.* **2010**, *66*, 125–132.
4. Winter, G.; Waterman, D. G.; Parkhurst, J. M.; Brewster, A. S.; Gildea, R. J.; Gerstel, M.; Fuentes-Montero, L.; Vollmar, M.; Michels-Clark, T.; Young, I. D.; Sauter N. K.; Evans G. DIALS: Implementation and evaluation of a new integration package. *Acta Crystallogr. Sect. D Struct. Biol.* **2018**, *74*, 85–97.
5. Evans, P. R.; Murshudov, G. N. How good are my data and what is the resolution? *Acta Crystallogr. Sect. D Biol. Crystallogr.* **2013**, *69*, 1204–1214.
6. Winn, M. D.; Ballard, C. C.; Cowtan, K. D.; Dodson, E. J.; Emsley, P.; Evans, P. R.; Keegan, R. M.; Krissinel, E. B.; Leslie, A. G. W.; McCoy, A.; McNicholas S. J.; Murshudov G. N.; Pannu N. S.; Potterton E. A.; Powell H. R.; Read R. J.; Vagin A.; Wilson K. S. Overview of the CCP4 suite and current developments. *Acta Crystallogr. Sect. D Biol. Crystallogr.* **2011**, *67*, 235–242.
7. McCoy, A. J.; Grosse-Kunstleve, R. W.; Adams, P. D.; Winn, M. D.; Storoni, L. C; Read, R. J. Phaser crystallographic software. *J. Appl. Crystallogr.* **2007**, *40*, 658–674.
8. Luo, D.; Xu, T.; Watson, R. P.; Scherer-Becker, D.; Sampath, A.; Jahnke, W.; Yeong, S. S.; Wang, C. H.; Lim, S. P.; Strongin, A.; Vasudevan S. G.; Lescar J. Insights into RNA unwinding and ATP hydrolysis by the flavivirus NS3 protein *EMBO J.* **2008**, *27*, 3209–3219.
9. Perrakis, A.; Harkiolaki, M.; Wilson, K. S.; Lamzin, V. S. ARP/wARP and molecular replacement. *Acta Crystallogr. Sect. D Biol. Crystallogr.* **2001**, *57*, 1445–1450.
10. Lebedev, A. A.; Young, P.; Isupov, M. N.; Moroz, O. V.; Vagin, A. A.; Murshudov, G. N. JLigand: A graphical tool for the CCP4 template-restraint library. *Acta Crystallogr. Sect. D Biol. Crystallogr.* **2012**, *68*, 431–440.
11. Jin, Y.; Molt, R. W.; Blackburn, G. M. Metal Fluorides: Tools for structural and computational analysis of

phosphoryl transfer enzymes. *Top. Curr. Chem.* **2017**, *375*, 1–31.

12. Griffin, J. L; Bowler, M. W.; Baxter, N. J.; Leigh, K. N.; Dannatt, H. R. W.; Hounslow, A. M.; Blackburn, G. M.; Webster, C. E.; Cliff, M. J.; Waltho, J. P. Near attack conformers dominate β-phosphoglucomutase complexes where geometry and charge distribution reflect those of substrate. *Proc. Natl. Acad. Sci.* **2012**, *109*, 6910–6915.
13. Chabre, M.; Aluminofluoride and beryllofluoride complexes: new phosphate analogs in enzymology. *Trends Biochem. Sci.* **1990**, *15*, 6–10.
14. Bock, C. W.; Kaufman, A.; Glusker, J. P. Coordination of water to magnesium cations. *Inorg. Chem.* **1994**, *33*, 419–427.
15. Smart, O. S.; Womack, T. O.; Sharff, A.; Flensburg, C.; Keller, P.; Paciorek, W.; Vonrhein, C.; Bricogne. G. Grade, version 1.2.12. **2011**, *Cambridge*, Global Phasing Ltd., http://www.globalphasing.com.
16. Potterton, E.; Briggs, P.; Turkenburg, M.; Dodson, E. A graphical user interface to the CCP4 program suite. *Acta Crystallogr. Sect. D Biol. Crystallogr.* **2003**, *59*, 1131–1137.
17. Emsley, P.; Lohkamp, B.; Scott, W. G.; Cowtan, K. Features and development of Coot. *Acta Crystallogr. Sect. D Biol. Crystallogr.* **2010**, *66*, 486–501.
18. Murshudov, G. N.; Skubák, P.; Lebedev, A. A.; Pannu, N. S.; Steiner, R. A.; Nicholls, R. A.; Winn, M. D.; Long, F.; Vagin, A. A. REFMAC5 for the refinement of macromolecular crystal structures. *Acta Crystallogr. Sect. D Biol. Crystallogr.* **2011**, *67*, 355–367.
19. Potterton, L.; Agirre, J.; Ballard, C.; Cowtan, K.; Dodson, E.; Evans, P. R.; Jenkins, H. T.; Keegan, R.; Krissinel, E.; Stevenson, K.; Lebedev A.; McNicholas S. J.; Nicholls R. A.; Noble M.; Pannu N. S.; Roth C.; Sheldrick G.; Skubak P.; Turkenburg J.; Uski V.; von Delft F.; Waterman D.; Wilson K.; Winn M.; Wojdyr M. CCP4i2: The new graphical user interface to the CCP 4 program suite. *Acta Crystallogr. Sect. D Struct. Biol.* **2018**, *74*, 68–84.
20. McNicholas, S.; Potterton, E.; Wilson, K. S.; Noble, M. E. M. Presenting your structures: The CCP4MG molecular-graphics software. *Acta Crystallogr. Sect. D Biol. Crystallogr.* **2011**, *67*, 386–394.
21. Schrödinger. The PyMOL Molecular Graphics System, Version 1.8. **2016**, V1.8.
22. Sievers, F.; Wilm, A.; Dineen, D.; Gibson, T. J.; Karplus, K.; Li, W.; Lopez, R.; McWilliam, H.; Remmert, M.; Söding, J.; Thompson, J. D.; Higgins, D. G. Fast, scalable generation of high-quality protein multiple sequence alignments using Clustal Omega. *Mol. Syst. Biol.* **2011**, *7*, 539–545.
23. Söding, J.; Biegert, A.; Lupas, A. N. The HHpred interactive server for protein homology detection and structure prediction. *Nucleic Acids Res.* **2005**, *33*, W244–W248.
24. Robert, X; Gouet, P. Deciphering key features in protein structures with the new ENDscript server. *Nucleic Acids Res.* **2014**, *42*, W320–W324.
25. Kohn, W.; Sham, L. J. Self-consistent equations including exchange and correlation effects. *Phys. Rev.* **1965**, *140*, A1133–A1138.
26. Hohenberg, P.; Kohn, W. Inhomogeneous electron gas. *Phys. Rev.* **1964**, *136*, B864–B871.
27. Zhao, Y.; Truhlar, D. G. The M06 suite of density functionals for main group thermochemistry, thermochemical kinetics, noncovalent interactions, excited states, and transition elements: Two new functionals and systematic testing of four M06-class functionals and 12 other function. *Theor. Chem. Acc.* **2008**, *120*, 215–241.
28. Mardirossian, N.; Head-Gordon, M. How accurate are the minnesota density functionals for noncovalent interactions, isomerization energies, thermochemistry, and barrier heights involving molecules composed of main-group elements? *J. Chem. Theory Comput.* **2016**, *12*, 4303–4325.
29. Jin, Y.; Molt, R. W.; Waltho, J. P.; Richards, N. G. J.; Blackburn, G. M. ^{19}F NMR and DFT analysis reveal structural and electronic transition state features for RhoA-catalyzed GTP hydrolysis. *Angew. Chemie Int. Ed.* **2016**, *55*, 3318–3322.
30. Jin, Y.; Molt, R. W.; Pellegrini, E.; Cliff, M. J.; Bowler, M. W.; Richards, N. G. J.; Blackburn, G. M.; Waltho, J. P. Assessing the influence of mutation on GTPase transition states by using X-ray Crystallography, ^{19}F

NMR, and DFT approaches. *Angew. Chemie Int. Ed.* **2017**, *56*, 9732–9735.

31. Mohr, P. J.; Newell, D. B.; Taylor, B. N. CODATA recommended values of the fundamental physical constants: 2014*. *Rev. Mod. Phys.* **2016**, *88*, 1–73.

32. Dunning, T. H. J. Gaussian basis sets for use in correlated molecular calculations. I. The atoms boron through neon and hydrogen. *J. Chem. Phys.* **1989**, *90*, 1007–1023.

33. Kendall, R. A.; Dunning, T. H. J. Electron affinities of the first-row atoms revisited. Systematic basis sets and wave functions. *J. Chem. Phys.* **1992**, *96*, 6796–6806.

34. Frisch, M. J.; Trucks, G. W.; Schlegel, H. B.; Scuseria, G. E.; Robb, M. A.; Cheeseman, J. R.; Scalmani, G.; Barone, V.; Mennucci, B.; Petersson, G. A.; *et al.* Gaussian 09, Revision E.01. **2009**, Gaussian, Inc., Wallingford CT.

35. Dennington, R.; Keith, T.; Millam, J. Gaussview. **2009**, Semichem Inc., Shawnee Mission K.

36. Schlegel, H. B. An efficient algorithm for calculating ab initio energy gradients using s, p Cartesian Gaussians. *J. Chem. Phys.* **1982**, *77*, 3676–3681.

37. Cramer, C. J. Essentials of computational chemistry: Theories and models. *2nd ed. John Wiley Sons, Ltd.* **2004**, West Sussex, U.K.

38. Sanner, M. F.; Olson, A. J.; Spehner, J. Reduced surface: An efficient way to compute molecular surfaces. *Biopolymers* **1996**, *30*, 305–320.

39. Pettersen, E. F.; Goddard, T. D.; Huang, C. C.; Couch, G. S.; Greenblatt, D. M.; Meng, E. C.; Ferrin, T. E. UCSF Chimera - A visualization system for exploratory research and analysis. *J. Comput. Chem.* **2004**, *25*, 1605–1612.

40. Jin, Y.; Bhattasali, D.; Pellegrini, E.; Forget, S. M.; Baxter, N. J.; Cliff, M. J.; Bowler, M. W.; Jakeman, D. L.; Blackburn, G. M.; Waltho, J. P. Fluorophosphonates reveal how a phosphomutase conserves transition state conformation over hexose recognition in its two-step reaction. *Proc. Natl. Acad. Sci.* **2014**, *111*, 12384–12389.

41. Baxter, N. J.; Bowler, M. W.; Alizadeh, T.; Cliff, M. J.; Hounslow, A. M.; Wu, B.; Berkowitz, D. B.; Williams, N. H.; Blackburn, G. M.; Waltho, J. P. Atomic details of near-transition state conformers for enzyme phosphoryl transfer revealed by MgF_3^- rather than by phosphoranes. *Proc. Natl. Acad. Sci.* **2010**, *107*, 4555–4560.

42. Ampaw, A.; Carroll, M.; Von Velsen, J.; Bhattasali, D.; Cohen, A.; Bowler, M. W.; Jakeman, D. L. Observing enzyme ternary transition state analogue complexes by ^{19}F NMR spectroscopy. *Chem. Sci.* **2017**, *8*, 8427–8434.

43. Cliff, M. J.; Bowler, M. W.; Varga, A.; Marston, J. P.; Szabó, J.; Hounslow, A. M.; Baxter, N. J.; Blackburn, G. M.; Vas, M.; Waltho, J. P. Transition state analogue structures of human phosphoglycerate kinase establish the importance of charge balance in catalysis. *J. Am. Chem. Soc.* **2010**, *132*, 6507–6516.

44. Uchikawa, E.; Lethier, M.; Malet, H.; Brunel, J.; Gerlier, D.; Cusack, S. Structural analysis of dsRNA binding to anti-viral pattern recognition receptors LGP2 and MDA5. *Mol. Cell* **2016**, *62*, 586–602.

45. Monecke, T.; Buschmann, J.; Neumann, P.; Wahle, E.; Ficner, R. Crystal structures of the novel cytosolic 5'-nucleotidase IIIB explain its preference for m7GMP. *PLoS One* **2014**, *9*, e90915–e90915.

46. Amin, E.; Jaiswal, M.; Derewenda, U.; Reis, K.; Nouri, K.; Koessmeier, K. T.; Aspenström, P.; Somlyo, A. V.; Dvorsky, R.; Ahmadian, M. R. Deciphering the molecular and functional basis of RhoGAP family proteins: A systematic approach toward selective inactivation of Rho family proteins. *J. Biol. Chem.* **2016**, *291*, 20353–20371.

47. Hemsworth, G. R.; González-Pacanowska, D.; Wilson, K. S. On the catalytic mechanism of dimeric dUTPases. *Biochem. J.* **2013**, *456*, 81–88.

48. Graham, D. L.; Lowe, P. N.; Grime, G. W.; Marsh, M.; Rittinger, K.; Smerdon, S. J.; Gamblin, S. J.; Eccleston, J. F. MgF_3^- as a transition state analog of phosphoryl transfer. *Chem. Biol.* **2002**, *9*, 375–381.

49. Wang, H.; Falck, J. R.; Hall, T. M. T.; Shears, S. B. Structural basis for an inositol pyrophosphate kinase surmounting phosphate crowding. *Nat. Chem. Biol.* **2011**, *8*, 111–116.

50. Bao, Z. Q.; Jacobsen, D. M.; Young, M. A. Briefly bound to activate: Transient binding of a second catalytic magnesium activates the structure and dynamics of CDK2 kinase for catalysis. *Structure* **2011**, *19*, 675–690.

51. Lee, J. Y.; Yang, W. UvrD helicase unwinds DNA one base pair at a time by a two-part power stroke. *Cell* **2006**, *127*, 1349–1360.

52. Yi, F.; Kong, R.; Ren, J.; Zhu, L.; Lou, J.; Wu, J. Y.; Feng, W. Noncanonical Myo9b-RhoGAP accelerates RhoA GTP hydrolysis by a dual-arginine-finger mechanism. *J. Mol. Biol.* **2016**, *428*, 3043–3057.

53. Yang, H.; Rudge, D. G.; Koos, J. D.; Vaidialingam, B.; Yang, H. J.; Pavletich, N. P. mTOR kinase structure, mechanism and regulation. *Nature* **2013**, *497*, 217–223.

54. Lau, W. C. Y.; Li, Y.; Liu, Z.; Gao, Y.; Zhang, Q.; Huen, M. S. Y. Structure of the human dimeric ATM kinase. *Cell Cycle* **2016**, *15*, 1117–1124.
